# Misdiagnosis: Acute Chest Syndrome That Evolved into Acute Respiratory Distress Syndrome in a Patient without a Documented History of Hemoglobinopathy

**DOI:** 10.1155/2019/2893056

**Published:** 2019-02-03

**Authors:** Christoph Sossou, Ogechukwu Chika-Nwosuh, Christopher Nnaoma, Jose Bustillo, Asad Chohan, Etinosasere Okundaye, Pratik Patel

**Affiliations:** ^1^Resident Physician, Internal Medicine, Newark Beth Israel Medical Center, Newark, NJ, USA; ^2^Associate Residency Program Director, Internal Medicine, Newark Beth Israel Medical Center, Newark, NJ, USA; ^3^Program Director, Pulmonary and Critical Care Fellowship, Newark Beth Israel Medical Center, Newark, NJ, USA

## Abstract

Acute chest syndrome (ACS) is a feared complication of sickle cell disease. Here is a case of a patient who presented with symptoms suggestive of acute chest syndrome yet had a delayed diagnosis presumably due to the lack of documented history of sickle cell disease of the patient, consequently evolving into acute respiratory distress syndrome (ARDS). He was subsequently diagnosed with heterozygous sickle cell SC disease on hemoglobin electrophoresis. After appropriate management with mechanical ventilator, broad-spectrum empiric intravenous antibiotics, exchange transfusion, and intravenous fluid resuscitation, the patient was medically optimized and safely discharged home, with significant improvement noted on successive follow-up visits.

## 1. Case Introduction

Sickle cell disease is remarkable for its clinical heterogeneity. Acute chest syndrome (ACS), characterized by new pulmonary infiltrates on chest radiography and one or more of these: cough, fever, hypoxia, chest pain, and shortness of breath, is a distinctive manifestation of sickle cell disease, most commonly associated with homozygous sickle cell disease (HbSS) and rarely with heterozygous sickle cell SC disease (HbSC) [1]. Herein, we present a case of a patient without documented history of hemoglobinopathy who presented with acute chest syndrome that rapidly evolved into acute respiratory distress syndrome presumably due to delayed diagnosis. In so doing, we aim to highlight the importance of not overlooking the possibility of sickle cell disease in Black or African American patients with acute pulmonary events who might not otherwise endorse a history of hemoglobinopathy.

## 2. Case Presentation

A 55-year-old African American male with a medical history of chronic nonvalvular atrial fibrillation, chronic coronary artery disease presented to the emergency department of our facility for evaluation of progressively worsening 2-day history of bilateral flanks and intermittent pleuritic chest pain with associated dyspnea, nonproductive cough, and tactile fever.

**Figure 1:**
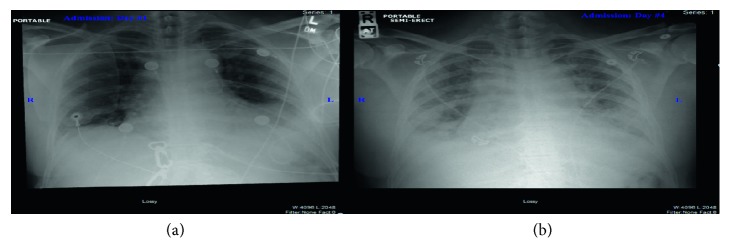
Portable chest radiography. (a) Defibrillator pad over the right chest wall. The lung is clear with sharp costophrenic angles bilaterally. Mild subsegmental atelectasis at the lung bases is seen. There are no airspace infiltrates, focal consolidation, pleural effusion, or pneumothorax. (b) Enlarged cardiomediastinal silhouette. Interval development of small bilateral pleural effusions and pulmonary congestion occurs.

**Figure 2:**
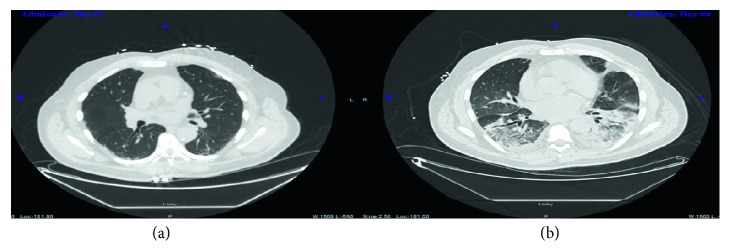
Contrast-enhanced chest computed tomography. (a) No filling defect in the main or lobar branches of the pulmonary arteries. Evidence of mild subsegmental atelectasis in the lung bases is seen. There are no airspace infiltrates, focal consolidation, or pleural effusion. (b) New development of bilateral basilar infiltrates and consolidations without pleural effusion.

On presentation, he was in mild distress, normotensive, tachycardic, tachypneic, febrile, and hypoxic (SpO_2_ 88% on room air). Cardiopulmonary exam revealed irregular heart rate, decreased breath sounds, and mild pulmonary rales in the bilateral lower lung bases. Otherwise, the rest of the exam was unremarkable. Initial laboratories revealed neutrophil-predominant leukocytosis, normochromic-normocytic anemia (hemoglobin 10 g/dL) with high reticulocyte index (12%), hyperbilirubinemia (total bilirubin 3 mg/dL and direct bilirubin 0.5 mg/dL), and negative troponin I. Chest radiography (CXR) and contrast-enhanced chest computed tomography (CT) showed no active disease (Figures 1(a) and 2(a)). The patient was admitted to the telemetry unit and managed for presumed diagnosis of community-acquired pneumonia. His condition subsequently worsened, he developed high-grade fever (T102°F), tachyarrhythmia (HR 120°BPM), and hypoxemia (SpO_2_∼70°s) on 4 liters nasal cannula oxygen with labored respiration necessitating endotracheal intubation and management with pressure-regulated volume control mechanical ventilator. Repeat imaging (CXR and chest CT) revealed extensive bilateral basilar airspace opacities with large evolving bilateral pleural effusions (Figures 1(b) and 2(b)), and arterial blood gas showed hypoxemia with arterial oxygen partial pressure to fractional inspired oxygen ratio (PaO₂ to FIO₂) of 88 mmHg. Hemoglobin electrophoresis revealed 0.0% hemoglobin A, 49.6% hemoglobin S, and 43.8% hemoglobin C. The patient was diagnosed with hemoglobin SC sickle cell disease complicated by acute chest syndrome which evolved into acute respiratory distress syndrome. He underwent urgent exchange transfusion. He was also managed with intravenous normal saline hydration at maintenance rate and broad-spectrum empiric IV antibiotics; his condition remarkably improved after 48 hours. He had an uneventful extubation and was safely discharged home after a total of 14-day hospital stay. Significant improvement was noted on subsequent follow-up visits.

## 3. Case Discussion

Approximately half of the patients with sickle cell disease (SCD) experience acute chest syndrome (ACS), and most of these cases occur with homozygous (SS) and infrequently with heterozygous (SC) sickle disease [2]. ACS is a lethal complication, characterized by new infiltrates on chest radiograph plus either dyspnea, chest discomfort, fever, or hypoxia [1]. Early stages can be difficult to recognize due to its clinical mimicry of other conditions (i.e., cardiogenic pulmonary edema, pneumonia, myocardial infarction, pulmonary infarction, etc.), possibly resulting in delay of critical interventions as was evident in our case [3]. HbSC is marked by milder clinical manifestations than HbSS disease, and the various complications of homozygous SS sickle cell disease (i.e., acute chest syndrome) occur irregularly or later in life [4]. In order words, while some suffer repeated crises requiring frequent hospitalization, a small percent of patients with HbSC disease remain virtually asymptomatic into or even through adult life. Due to this fact, some of these asymptomatic patients would usually go about their lives thinking they do not have the disease. Additionally, even though the Sickle Cell Anemia Control Act was enacted in 1972, it was not until May 2006 that all states within the United States of America required and provided universal newborn screening for SCD despite a national recommendation to this effect in 1987 [5]. Our patient was born in 1962; it is reasonable to assume he was never screened or if he was screened, he might have forgotten he had the disease since he has been asymptomatic his entire adult life. This might have led him to deny a history of having a sickle cell disease. Owing to its heterogeneous clinical manifestations [6], HbSC is often overlooked as the culprit of acute pulmonary events in adult patients who denied history of hemoglobinopathy. One important issue highlighted in this case is that history and a single physical examination or radiograph may not be adequate for early diagnosis of acute chest syndrome secondary to HbSC; hence, it is paramount that clinicians maintain a high index of suspicion, especially in Blacks or African American patients, as the prevalence of SCD (HbSS or HbSC) is high in this population [7]. One of the feared complications of acute chest syndrome is rapid progression to acute respiratory distress syndrome (ARDS), an exceedingly rare, yet often fatal complication. ARDS is defined by the acute onset (≤1 week) of bilateral opacities on chest imaging that are not fully explained by cardiogenic pulmonary edema, physiologic shunt requiring positive end-expiratory pressure, and Pa2/FiO2 ratio <300 mmHg [8]. The morbidity and mortality rate of ARDS is traditionally very high, but recent advancements in patient care have significantly reduced both morbidity and mortality rates [8–11]. Prompt recognition of ARDS is of absolute importance as this would ensure timely interventions are undertaken. The mechanism by which acute chest syndrome evolves into acute respiratory distress syndrome is yet to be elucidated. There are few case reports in the literature where known sickle cell disease complicated by acute chest syndrome evolved into acute respiratory distress syndrome [12–14], but our case is the first known case in the literature where the patient's sickle cell disease status was unknown. This case also highlights the need to consider a broad array of differential diagnoses in patients, particularly Black or African American patients presenting with acute pulmonary events. There are no official data on the number of people who were not screened at birth for hemoglobinopathy prior to the implementation of the required universal newborn screening in 2006; hence, it is paramount that clinicians maintain a high index of suspicion when caring for the population of patients with high prevalence of SCD.
